# CDK4/6 inhibition stabilizes disease in patients with p16-null non-small cell lung cancer and is synergistic with mTOR inhibition

**DOI:** 10.18632/oncotarget.26424

**Published:** 2018-12-21

**Authors:** Priya K. Gopalan, Andres Gordillo Villegas, Chunxia Cao, Mary Pinder-Schenck, Alberto Chiappori, Wei Hou, Maria Zajac-Kaye, Alison M. Ivey, Frederic J. Kaye

**Affiliations:** ^1^ Department of Medicine, University of Florida, Gainesville, FL, USA; ^2^ Moffitt Cancer Center, Tampa, FL, USA; ^3^ Department of Biostatistics, University of Florida, Gainesville, FL, USA; ^4^ Department of Anatomy and Cell Biology, University of Florida, Gainesville, FL, USA; ^5^ Current address: Sangamo Therapeutics, Richmond, CA, USA; ^6^ Current address: Merck, Philadelphia, PA, USA; ^7^ Current address: Division of Epidemiology and Biostatistics, Stony Brook University, Stony Brook, NY, USA

**Keywords:** non-small cell lung cancer, CDK 4/6, palbociclib, mTOR, clinical trial

## Abstract

Aberrant activation of CDK4/6 kinase is the most common somatic event in non-small cell lung cancer (NSCLC). Palbociclib is a highly specific CDK4/6 inhibitor shown to inhibit cell cycle progression and promote cellular senescence. We conducted a phase 2 clinical trial of palbociclib in 19 previously-treated patients with advanced NSCLC. Only patients with p16-null staining by immunohistochemistry and documented tumor progression were eligible. The primary endpoint was tumor response rate. Palbociclib therapy alone was well-tolerated. Of 16 evaluable patients who received > 1 month of therapy, there were no objective responses. However, 8 patients (50%) with previously progressive NSCLC had stable disease (SD) lasting a range of 4-10.5 months. Median overall survival (OS) for all cases was 5.1 months, and median overall survival for the subset of patients with SD was 16.6 months. We also performed preclinical testing of palbociclib in combination with 13 different targeted or cytotoxic chemotherapeutic agents using a cell viability assay. Only the combination of palbociclib and mTOR inhibitors resulted in synergistic growth inhibition, particularly in tumors carrying RAS mutations. Our findings warrant further clinical investigation of the combination of palbociclib and mTOR inhibitors, especially in patients carrying activated RAS mutations.

## INTRODUCTION

Lung cancer is the leading cause of cancer mortality in the United States [1] and worldwide [2] despite the promise of new therapeutic strategies, survival remains poor for most patients with advanced disease [3]. The epistatic retinoblastoma (RB: CDK4/6: Cyclin D: CDKN2a/p16) signaling pathway is targeted for somatic or epigenetic alterations in essentially 100% of lung cancer samples [4, 5]. In the case of small cell lung cancer (SCLC), almost 90% of tumors specifically target the RB gene with loss-of-function somatic mutations, and the remaining tumors show genetic or epigenetic alterations in either CDKN2a/p16 or other cryptic gene loci within the RB tumor suppressor pathway [6, 7]. In contrast, non-small cell lung cancer (NSCLC) exhibits a different genetic landscape where at least 90% of lung squamous cell carcinomas and lung adenocarcinomas retain a functional RB gene product. Studies from different research groups worldwide have shown that the majority of NSCLC tumors preferentially target the CDKN2a/p16 locus for mutational or epigenetic loss of function [4, 5, 8–11]. In normal cells, the CDKN2a/p16 gene product binds and inactivates CDK4/6:cyclin D kinase in the absence of growth factor signals to maintain a resting or quiescent cellular state. In contrast, somatic tumor-specific loss of CDKN2a/p16 results in constitutive CDK4/6 signaling associated with aberrant RB hyperphosphorylation. This post-translational event induces a conformation change in the RB binding pocket releasing nuclear binding proteins that trigger a cascade of transcriptional events driving cells through the G1/S phase of the cell cycle toward DNA replication [5]. Although hundreds of gene products are required for the efficient entry and exit of the cell cycle, tumor cells have surprisingly targeted a limited number of cell cycle regulated genes that cluster epistatically with the RB signaling pathway at the G1/S transition, suggesting a highly attractive therapeutic target [5, 11–15].

Palbociclib is a highly specific CDK4/6 inhibitor that has been shown to inhibit cell cycle progression and promote cellular senescence *in vitro* with nanomolar IC_50_'s [16, 17]. Remarkably, it was predicted over 2 decades ago that CDK4/6 inhibition would only restrain tumor cell proliferation in samples that retained an intact functional RB gene product. Preclinical data using palbociclib confirmed this prediction with tumor cell growth inhibition exclusively in cancer cells that retained wildtype RB protein [16]. CDK 4/6 inhibitors have been approved by the FDA for use in combination with anti-hormone agents in breast cancer. Since most common adult tumors retain wildtype RB function, participants in breast cancer clinical trials were not selected by RB/CDKN2A status but solely by ER status. Patients with ER-positive/HER2-negative breast cancer experienced significantly improved the median progression-free survival (PFS) when taking palbociclib combined with letrozole compared to letrozole alone (24.8 months vs 14.5 months, HR 0.58, *p* < 0.001) [18]. To date, more than 200 clinical trials with CDK4/6 inhibitors have been completed or are underway in many different disease types, including breast cancer, mantle cell lymphoma, liposarcoma, NSCLC, glioblastoma multiforme, germ cell tumors, melanoma and SCLC [8, 17, 19, 20].

There have been a several clinical trials with CDK 4/6 inhibitors in patients with NSCLC. In one phase 1 clinical trial with expansion cohorts in several different primary tumors, 68 molecularly unselected patients with previously-treated advanced NSCLC were treated with the CDK4/6 inhibitor, abemaciclib [21]. The disease control rate (DCR) was 49%, where 2 patients showed a partial response (one of whom was p16 null) and 31 patients had stable disease. Furthermore, 15 patients (22%) achieved stable disease for at least 24 weeks, and 4 patients had stable disease for at least 12 months. The Lung-MAP clinical trial had one cohort of patients with previously-treated stage IV squamous cell carcinoma of the lung who were randomized to treatment with either palbociclib or docetaxel (study S1400C) [22]. Patients were required to have a CDK4 mutation or cyclin gene family CCND1, D2 or D3 amplification. A total of 54 patients were registered, of whom 32 received palbociclib. This arm of the trial using single agent therapy was closed due to futility. However, the DCR was 44%, where 12 patients treated with palbociclib had stable disease and 2 patients with a documented partial response had a CCND1 abnormality [22].

We now report results of a phase 2 clinical trial studying the efficacy of palbociclib alone in patients with previously-treated advanced NSCLC who were preselected for CDKN2a/p16 loss using immunohistochemistry and who had radiographic evidence for documented tumor progression at enrollment. We have also pursued preclinical testing with combination drug therapy to identify additive or synergistic relationships between CDK 4/6 inhibition and the inhibition of other key cancer gene pathways, with a goal of optimizing tumor response.

## RESULTS

### Palbociclib treatment in heavily pre-treated patients with advanced NSCLC

Twenty-five patients with previously treated advanced stage NSCLC, with documented p16-null status by immunohistochemistry, and with documented tumor progression on CT imaging using RECISTv1.1 [23] were enrolled onto a phase 2 clinical trial from April 2012 to June 2013. A total of 25 patients were consented (Supplementary Figure 1). Six patients did not receive study drug due to either withdrawal of consent (*n* = 3), the subsequent presence of p16 staining by immunohistochemistry (*n* = 1), or failure to meet other eligibility criteria (*n* = 2). Nineteen patients started palbociclib, and were therefore evaluable for toxicity. Eleven of 19 patients had adenocarcinoma and 8 patients had squamous cell carcinoma (Supplementary Table 1). Six patients had Eastern Cooperative Oncology Group performance status [24] (ECOG PS) 0, 12 patients had ECOG PS 1, and 1 patient had ECOG PS 2. The patients received an average of two prior lines of cytotoxic chemotherapy treatment (range 1-4 lines of treatment) prior to enrolling in the study. Palbociclib at 125 mg was taken orally daily on days 1-21 of a 28-day cycle. Three patients received less than one cycle (28 days) of drug, because they elected to discontinue treatment and enroll in hospice, and were not evaluable for response. Therefore, a total of 16 patients were available for analysis of response.

Single-agent palbociclib therapy was well-tolerated (Table 1). One patient experienced multiple grade 3 and 4 toxicities as a result of hepatitis and rhabdomyolysis, with a maximum CPK level of > 20,000 units/L, which were related to concomitant use of high-dose simvastatin (80 mg daily). The same patient experienced grade 3 thrombocytopenia and grade 4 neutropenia. Two other patients developed grade 3 neutropenia, but neither developed fever or required hospitalization. All other toxicities were mild or moderate in severity (grades 1 or 2).

**Table 1:** Adverse events caused by palbociclib

	Grade 1	Grade 2	Grade 3	Grade 4
**Gastrointestinal**	16	7	0	0
**HEENT**	14	4	0	0
**Constitutional**	8	7	0	0
**Hematologic**				
Neutropenia	1	2	2	1^†^
Thrombocytopenia	2	1	1^†^	0
Anemia	1	3	0	0
**Musculoskeletal/Pain**	9	2	1^†^	0
**Pulmonary**	7	2	0	0
**Neurological**	7	0	0	0
**Dermatologic**	5	1	0	0
**Metabolic/Laboratory**	1	0	1^†^	2^†^
**Others**	6	3	0	0

^†^Adverse events in a single patient on high-dose simvastatin

We performed an intention-to-treat analysis of the eligible 16 heavily pretreated patients who received the study drug for at least 1 month. Eight patients (50%) experienced stable disease (SD), lasting 4-10.5 months, of whom six patients had SD lasting ≥6 months (Table 2). The median PFS was 3.2 months (95% CI: 2.1-6.0 months) for all 16 patients, 6.1 months (95% CI: 4.0-10.0 months) for the 8 patients with SD, and 2.2 months (95% CI: 1.0-2.4 months) for the 8 patients with progressive disease (PD) (log-rank *p* value < 0.0001) (Table 2, Supplementary Table 2).

**Table 2:** Characteristics and response to treatment in patients with stable disease greater than 2 months

Age	Gender	Ethnicity/Race	Performance Status	Histology	# prior lines of therapy	EGFR status	KRAS status	ALK status	Best Response	Duration of SD (months)	OS (months)
64	F	Caucasian	1	Adeno	1	WT	WT	WT	SD	10.5	34.9
72	F	Caucasian	1	Adeno	1	WT	WT	WT	SD	10	19.5
69	M	Caucasian	1	Adeno	2	WT	WT	WT	SD	8	22.5
77	M	Caucasian	1	Squamous	2	WT	WT	ND*	SD	6.25	33.4
74	M	Caucasian	1	Adeno	3	WT	WT	WT	SD	6	8
63	M	Hispanic	1	Adeno	3	WT	ND	ND	SD	6	9.25
71	F	Caucasian	1	Squamous	3	ND	ND	ND	SD	4.25	5.75
68	F	African American	0	Squamous	3	ND	ND	ND	SD	4	13.5

F, female; M, male; WT, wildtype; ND, not determined; SD, stable disease; OS, overall survival

We were able to extract DNA and perform an exploratory analysis of the cancer gene mutational status on archival biopsy samples from 11 of 16 patients and did not detect EGFR, KRAS or ALK gene alterations in any cases (Table 2, Supplementary Table 2). Therefore, we could not assess the potential preferential benefit of palbociclib in patients with KRAS mutant lung cancer.

Although the trial design did not allow us to make conclusions about the effect of palbociclib on overall survival (OS), we were able to assess the 16 patients for survival. The median OS for the evaluable population was 7.7 months (95% CI: 4.0-13.5 months), and the median OS for the subset of patients with and without SD were 16.5 months (95% CI: 5.8-33.4 months) and 4.2 months (95% CI: 2.5-7.6 months), respectively (log-rank *p* = 0.0003). In summary, we observed a high rate of disease stability consistent with preclinical data and reports from other clinical trials which demonstrate that palbociclib alone acts predominantly as a cytostatic agent and induces senescence but not apoptosis [25–27].

### Studies of combination therapy with palbociclib in NSCLC

**Figure 1:**
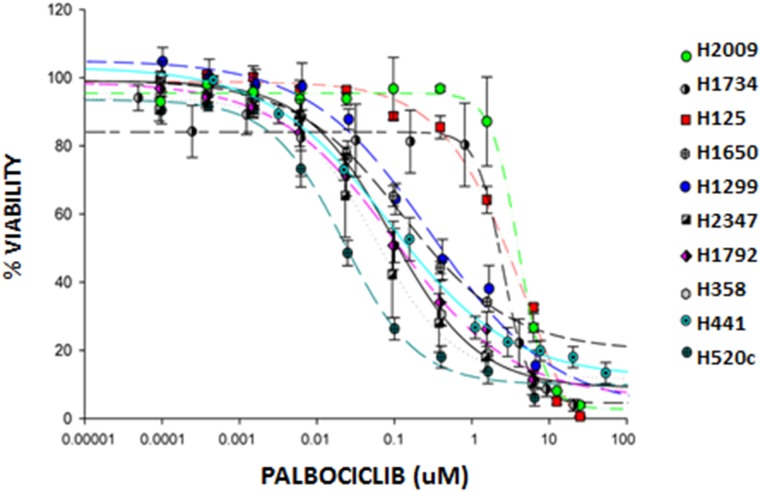
Palbociclib inhibits growth of NSCLC cell lines NSCLC cell lines were treated with increasing doses of palbociclib for 7 days. The Cell Titer-Blue® Cell Viability Assay was used to quantitate cell metabolic activity as a surrogate marker for viability. Viability was expressed as a percent of untreated cells. Data represent the mean ± SEM of at least three independent experiments, each performed in triplicate.

Since we observed in our Phase 2 clinical trial that single-agent palbociclib led to stable disease in 50% of heavily pre-treated patients with progressive NSCLC with no objective tumor responses, we recognized that combination drug therapy will be required to achieve meaningful clinical benefit to this large subgroup of CDKN2a/p16-negative NSCLC patients. To develop new strategies to optimize palbociclib-mediated tumor growth inhibition in lung cancer, we tested the effects of palbociclib in 13 different annotated NSCLC cell lines with absent p16 expression (validated CKDN2A somatic mutations or hypermethylation), and in two tumor cell lines with RB-null, p16-intact expression which were included as negative control for CDK4/6 inhibition (H1734 and H2009) (Figure 1 and data not shown). While we detected a range of cell growth inhibitory responses to palbociclib in cell lines from different patients with absent p16 expression, we observed, as predicted, that the RB-null H1734 and H2009 cell lines showed some of the greatest relative drug resistance to palbociclib (Figure 1, Table 3).

**Table 3:** IC_50_ values for palbociclib treatment and mutational profiles of NSCLC cell lines

			GENETIC STATUS										
CELL LINE	HISTOLOGY	PALBOCICLIB IC^50^ (nM)	p16	RB	CDKN2A	APC	EGFR	KIT	KRAS	HRAS	NRAS	PDGFRA	TP53
H520	SQ	21.6 ± 3.7	-	+	DELETED	WT	WT	WT	WT	WT	WT	WT	MT
H441	AD	62.4 ± 10	-	+	METHYLATED	WT	WT	WT	MT	WT	WT	WT	MT
H358	BAC	87.8 ± 16	-	+	METHYLATED	WT	WT	WT	MT	WT	WT	WT	WT
H1792	AD	100.2 ± 45	-	+	METHYLATED	WT	WT	WT	MT	WT	WT	WT	MT
H2347	NOS	134 ± 22	-	+	METHYLATED	WT	WT	WT	WT	WT	MT	WT	MT
H1299	LCC	283 ± 220	-	+	DELETED	WT	WT	WT	WT	WT	MT	WT	MT
H1650	BAC	730± 240	-	+	DELETED	WT	MT	WT	WT	WT	WT	WT	MT
H125	AD	5421 ± 1606	-	+	DELETED	WT	WT	WT	WT	WT	WT	WT	MT
H1734	NOS	3864 ± 464	+	-	UNMETHYLATED	WT	WT	WT	MT	WT	WT	WT	MT
H2009	AD	4086 ± 384	+	-	UNMETHYLATED	WT	WT	WT	MT	WT	WT	WT	MT

The dashed horizontal lines divide the p16 absent cell lines into different groups based on sensitivity to palbociclib. p16-absent cell lines in the top group are highly sensitive to palbociclib. The cells in the middle group have intermediate sensitivity, and the cell lines in the lower group are considered non-responders. IC_50_ values are the mean ± SEM of at least three independent experiments, each performed in triplicate. SQ, squamous cell carcinoma; AD, adenocarcinoma; BAC, bronchioalveolar carcinoma (adenocarcinoma-in-situ); NOS, not otherwise specified; LCC, large cell carcinoma; WT, wildtype; MT, mutated.

We analyzed and annotated the mutational profiles of the lung tumor cell lines to determine molecular marker(s) that might predict response to palbociclib (Table 3). We observed that p16-null lung tumor cells with the lowest IC_50_ typically had KRAS, NRAS or HRAS mutations, suggesting enhanced palbociclib sensitivity in the presence of a RAS mutation and absence of p16 expression as previously proposed [25]. In contrast, loss of RB expression was dominant over concurrent mutant KRAS to render those cell lines insensitive to palbociclib. These data further support the hypothesis that RB is the key or sole substrate for CDK4/6:cyclin D phosphorylation and emphasizes the importance of molecular selection of lung cancers that retain functional RB expression. Our data also suggest two categories of palbociclib resistant tumors. The first class of palbociclib non-responders represents the well-documented cases with RB null status. A second class of palbociclib non-responders comprise tumor samples with RB wildtype/p16 null status and concurrent wildtype RAS, however a mechanism underlying this palbociclib-resistance subset has not been defined.

We, therefore, recognized that combination drug therapy would be required to achieve meaningful clinical benefit with palbociclib therapy. To identify therapeutic agents that may synergize with CDK4/6 inhibition to induce cytotoxicity, we tested 15 compounds in the H358 cell line (Table 4 and data not shown). We were specifically interested in testing three strategies to enhance palbociclib cytotoxicity. First, since hypermethylation of CDKN2A with loss of p16 expression is the common mechanism to activate CDK4/6 in NSCLC [4, 28], we tested decitabine, a potent demethylating agent that can efficiently induce p16 protein expression to block CDK4/6 activity independently of exposure to palbociclib [4]. Therefore, we anticipated that the combination of demethylating the CDKN2A locus to reactivate p16-mediated CDK4/6 inhibition with palbociclib-mediated CDK4/6 inhibition would target dual independent mechanisms to block cell growth more effectively than palbociclib alone, particularly in cell lines with hypermethylated CDKN2A. Second, we anticipated that combining MEK and CDK4/6 inhibition would enhance tumor cell cytotoxicity *in vitro* [25, 29–31] and we studied these combinations in cell lines with either wildtype or mutant RAS. Finally, we were aware of potential compensatory feedback induction of CDK2:cyclin A or CDK2:cyclin E kinase activity following chronic CDK4/6 inhibition [32–34]. Therefore, we were interested in studying the ability of combined CDK2 and CDK4/6 inhibition to overcome potential drug resistance. We therefore tested 5 different anti-tumor compounds, including decitabine (demethylating), depsipeptide (histone deacetylase inhibitor), selumitinib (MEK inhibitor), and AZD 5438 and flavopiridol (multi-CDK inhibitors) (Table 4). However, using the CalcuSyn combination index (CI) [35], we did not detect drug synergy with any of these agents in combination with palbociclib.

**Table 4:** Effect of palbociclib in combination with various therapeutic agents

TARGETED/CHEMOTHERAPEUTIC AGENTS	% VIABILITY			
	PALBOCICLIB	AGENT	COMBINATION AGENT/PALBOCICLIB	CI
SELUMITINIB	51.7	85.2	58.7	2.28
DECITABINE	45.01	71.3	47.66	1.25
FLAVOPIRIDOL	45.3	7.5	9.8	1.09
SUNITINIB	41	77.4	40.39	0.9
AZD5438	50.54	85.74	50.6	0.86
PONATINIB	58.43	69.8	33.05	0.75
AZD4547	60.21	66.79	40.28	0.69
ERLOTINIB	50.77	84.94	43.9	0.66
PEMETREXED	54.24	13.55	12.8	0.6
PF04691502	57.45	50.94	18.75	0.5
EVEROLIMUS	58.82	32.53	18.82	0.26
DACOMITINIB	52.9	60.6	30.15	0.14
RAPAMYCIN	50.81	34.4	18.43	<0.1

H358 cells were treated for 72 hours with palbociclib (2.5 μM) and/or selumetinib (9.7 nM), decitabine (9.7 nM), flavopiridol (0.780 μM), sunitinib (97nM), and AZD5438 (9.7 nM). Cells were also treated for 7 days with palbociclib (97 nM) and/or ponatinib (97 nM), AZD 4547 (1.56 μM), erlotinib (9.7 nM), pemetrexed (97 nM), PF04691502 (0.195 μM), everolimus (9.7 nM), dacomitinib (12 nM), and rapamycin (9.7 nM). All concentrations used were at or near the IC50 doses. Viability was expressed as a percent of untreated cells. Data represent the mean ± SEM of at least three independent experiments, each performed in triplicate.

We also studied the effect of commercially-available small molecule inhibitors, including the multi-targeted tyrosine kinase inhibitors sunitinib, sorafenib, pazopanib and ponatinib, and did not detect drug synergy with palbociclib for tumor cell growth inhibition (Table 4 and data not shown). We also studied pemetrexed, a commercially available chemotherapeutic agent used for patients with advanced lung adenocarcinoma. Since pemetrexed, a folate antagonist, depends on cell proliferation to exert cytotoxic activity, we tested different drug treatment doses and schedules including: pre-incubation with palbociclib with 24- and 48-hour washout periods prior to adding pemetrexed, concurrent application of both agents and sequential incubation with pemetrexed first followed by 5-day exposure to palbociclib (data not shown), with no significant additive or synergistic effect over exposure to pemetrexed alone. These data are consistent with the hypothesis that palbociclib induces senescence which limits the cytotoxic activity of cancer agents that rely on cell proliferation [36, 37].

**Figure 2:**
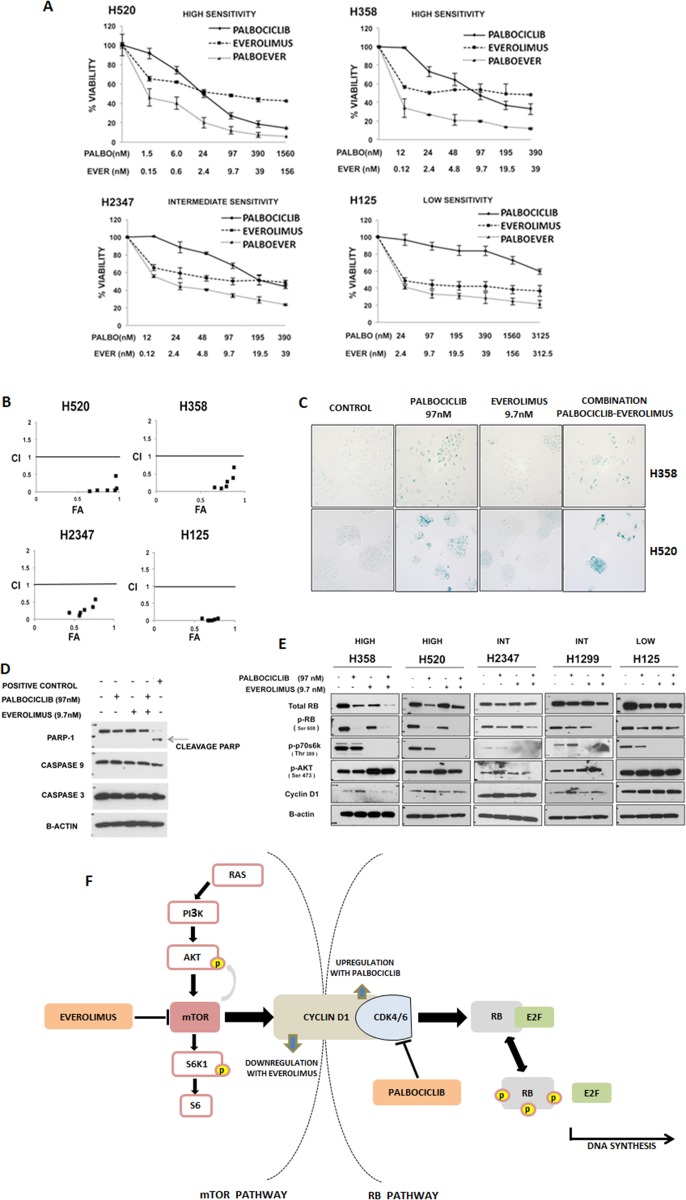
Targeting the RB and mTOR pathways synergistically inhibits NSCLC **A.** NSCLC cell lines that were highly sensitive (“SENSITIVE”), moderately sensitivity (“INTERMEDIATE”) and resistant (“RESISTANT”) to palbociclib were treated for 7 days with palbociclib and everolimus alone and in combination. For the combination, palbociclib and everolimus were combined in a fixed 1:10 ratio while changing the concentrations. Viability was expressed as a percent of untreated cells. Data represent the mean ± SEM of at least three independent experiments, each performed in triplicate. **B.** CalcuSyn graph of combination index values (CI) *vs* fraction affected (FA) for the cell lines indicated, based on data presented in figure 2A. A CI value less than 0.8 is considered synergistic. **C.** β-galactosidase staining of H358 and H520 cell lines to determine senescence. Cells were treated for 7 days with palbociclib (97 nM) and/or everolimus (9.7 nM). Bright field pictures (100X magnification) are representative of at least three independent experiments. **D.** H358 cells were treated with palbociclib (97 nM) and/or everolimus (9.7 nM) for 24 hours, and their effect on PARP-1 protein and caspases 3 and 9 was determined by immunoblot analysis. The positive control is a lysate of BON cells treated with 10µM of PF04554878. Each blot is representative of two independent experiments. **E.** Immunoblot analyses of total RB, phosphorylated RB (pRB), phosphorylated p70S6K (pp70S6K), phosphorylated AKT (pAKT), cyclin D1 and β-actin after 24 hours of treatment with palbociclib at 97 nM and/or everolimus at 9.7 nM in NSCLC cell lines highly sensitive (“HIGH”), moderately or intermediate sensitive (“INT”), and with low sensitivity/resistant (“LOW”) to palbociclib. Each blot is representative of at least two experiments. **F.** Diagram of Cyclin D1 as a link between the mTOR and RB pathways. The inhibition of CDK4/6 by palbociclib leads to a compensatory increase in the levels of cyclinD1. This increase in turn affects the AKT/mTOR pathway by leading to downregulation of phospho-p70S6K. Everolimus blocks CyclinD1 induction following palbociclib treatment.

We also studied palbociclib combinations with the EGFR inhibitors erlotinib and dacomitinib (a pan-EGFR inhibitor), and the mTOR inhibitors rapamycin, everolimus and PF04691502 (a dual PI3K/mTOR inhibitor) (Table 4). We observed the greatest degree of cell growth inhibition with each of the mTOR inhibitors, rapamycin, everolimus and PF 04691502. To validate the detection of enhanced growth inhibition observed with palbociclib in combination with mTOR pathway inhibitors, we studied the combination of palbociclib with everolimus, rapamycin and PF04691502 in three additional p16-null lung tumor cell lines and confirmed synergy (Figure 2A and 2B, Supplementary Figures 2 and 3).

Since palbociclib inhibits cell proliferation via induction of senescence [36–38], we were interested in assessing if the growth inhibition observed with the combination of palbociclib and the mTOR inhibitor everolimus was associated with enhancement of senescence or induction of apoptotic cell death. We utilized a β-galactosidase assay to assess senescence in the p16-null cell lines, H358 and H520 (Figure 2C). In both cell lines, palbociclib significantly increased senescence over control cells. Cells exposed to everolimus alone did not exhibit detectable markers for senescence and the drug combination did not appear to increase β-galactosidase expression. Immunoblot analysis of H358 cells exposed to the combination of palbociclib and everolimus did not show an increase in caspase 3 or 9, or PARP-mediated cleavage products over cells treated with palbociclib alone, suggesting that apoptosis is not induced by combined drug treatments (Figure 2D). While combined therapy showed synergistic growth inhibition, these data suggest that everolimus does not significantly enhance measurements of apoptosis or cell senescence compared to palbociclib alone.

To further study the effects of the combination of CDK4/6 and mTOR inhibition, we performed protein immunoblot analysis on p16-null NSCLC cells exposed to both drugs (Figure 2E). We confirmed that exposure to mTOR inhibitors abrogated p70S6K phosphorylation, which was not affected by palbociclib alone. We also detected the expected compensatory increase in pAKT expression, which was also unaffected by palbociclib (either alone or combined with everolimus). Interestingly, we observed a compensatory upregulation of cyclin D1 following palbociclib-mediated CDK4/6 inhibition. Since cyclin D1 is normally induced by mTOR activation [39, 40], cyclin D1 expression decreases following mTOR inhibition. We now show that palbociclib combined with everolimus might function to overcome a compensatory upregulation of cyclin D1 with palbociclib therapy alone (Figure 2E, Figure 2F).

**Figure 3:**
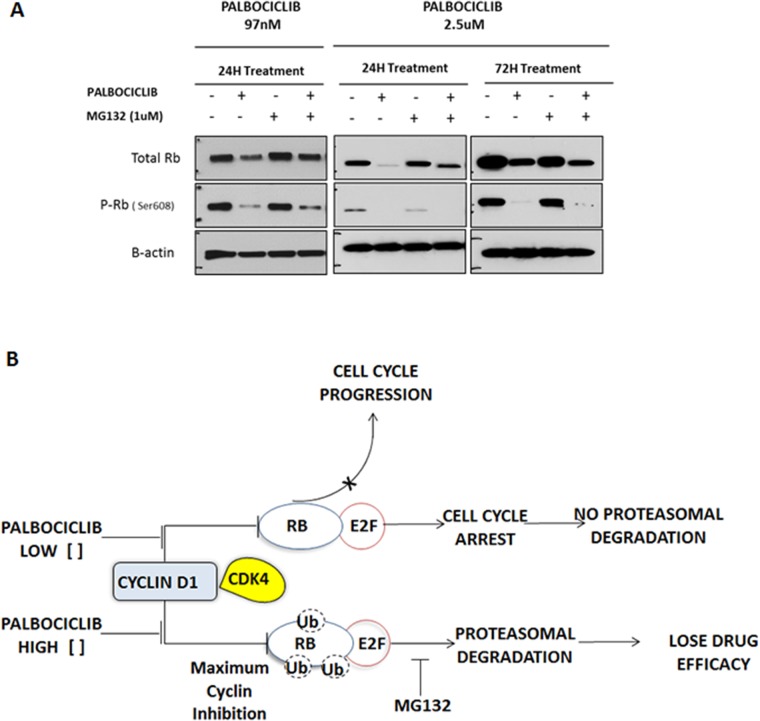
Effect of high-dose palbociclib on total RB **A.** H358 NSCLC cells were treated for 24 and 72 hours with palbociclib (97 nM and 2.5 µM) and/or the proteasome inhibitor MG132 (1.0 µM), and their effect on total RB protein and phosphorylated RB (P-RB) was determined by immunoblot analysis. Each blot is representative of two independent experiments. **B.** Postulated mechanism of the dosage effect of palbociclib on total RB concentrations. At low concentrations of palbociclib, inhibition of CDK4/6 leads to selective inhibition of RB phosphorylation. A high concentration of palbociclib was associated with proteasomal degradation of total RB.

To confirm the efficiency of palbociclib-mediated CDK4/6 inhibition, we studied phosphorylated RB (phospho-RB) protein by immunoblot analysis after drug exposure (Figure 3A). As expected, we detected a reduction of phospho-RB, however we also detected a decrease in total pan-RB expression as well. Therefore, we were concerned that much of the decrease in phospho-RB could be a non-specific toxic effect of palbociclib on total pan-RB that would be deleterious for tumor growth inhibition *in vitro* and for tumor response *in vivo*. We noted that higher doses of palbociclib (2.5 µM) markedly decreased levels of total pan-RB after 24 and 72 hours incubation (Figure 3A). There are several possible reasons, including degradation of RB (via proteasome pathway) and/or apoptosis of cells exposed to palbociclib. In order to explore the first possibility, we observed that 1 µM MG132, a proteasome inhibitor, was able to partially rescue palbociclib-mediated decrease in total RB, particularly at lower palbociclib doses where we can detect a preferential decrease in phosphorylated RB with protection of total RB protein levels. We observed no change in PARP-1 cleavage, or caspase 9 or caspase 3 levels, suggesting no role of palbociclib in apoptosis (Figure 2D). Thus, it appears that high palbociclib concentrations are associated with non-specific proteosomal degradation of RB (Figure 3B) that may be deleterious in treatment of patients in clinical trials. In contrast, with lower palbociclib concentrations, steady-state RB protein levels are stable, showing specific inhibition of RB phosphorylation linked to tumor cell senescence.

## DISCUSSION

Targeting CDK4/6 has been an attractive goal for more than 20 years since the identification of the RB:CDK4:cyclin D:p16 pathway. However, early trials targeting CDKs were unsuccessful (19)], perhaps due to inefficient drug access to a critical nuclear subcellular compartment or incomplete kinase inactivation. The identification of a potent and highly specific CDK4/6 inhibitor, palbociclib, has renewed interest in CDK4/6 inhibition and there are now more than 200 ongoing or completed clinical trials using CDK4/6 inhibitors.

In our clinical trial, we used absence of p16 protein staining by immunohistochemistry as a requirement for eligibility, rather than detection of RB staining, for several reasons. First, loss of CDKN2A/p16 is the most common somatic genetic or epigenetic event in NSCLC and is mutually exclusive with loss of RB function in lung cancer [41, 42]. In addition, RB staining is often focal within biopsy sections [43] and may be hard to validate with the smaller biopsies and cytology samples obtained in lung cancer patients. Further, rare RB missense mutations or integration of DNA tumor viral sequences can occasionally inactivate RB protein function without affecting steady-state levels [44, 45]. Finally, loss of RB function is associated with compensatory induction of steady-state p16 protein levels [46], and therefore, we were able to use the same CLIA-certified test for p16 that is performed as a surrogate of HPV activity in cervical and oropharyngeal cancers. This test is available in many hospitals, with rapid turnaround times.

Interestingly, molecular selection by p16 or RB status for this drug may not be necessary. In the Lung-MAP clinical trial, patients with advanced lung squamous cell carcinoma with CDK4 or CCND1/2/3 amplifications, which are molecularly similar to the p16-null state, were randomized to palbociclib or docetaxel. The DCR of 44% and PFS of 1.7 months was similar to what we observed. In another clinical trial, the CDK4/6 inhibitor, abemaciclib, was given to 49 molecularly unselected patients with advanced NSCLC, with DCR of 51% and median PFS of 2.1 months, which were comparable to our molecularly selected population (DCR 50%, median PFS 3.2 months) [47]. The similar results are likely due to the low rate of RB-null NSCLC tumors, estimated at only 10% [41].

A potential molecular biomarker for palbociclib response is mutant KRAS status, first suggested through *in vitro* and *in vivo* work in tumor cell lines and mice where synthetic lethality was observed following CDK4 inhibition [25, 48]. In addition, the DCR was reported to be higher in the subset of patients with KRAS mutation (DCR 54%) compared to KRAS wildtype (DCR 37%) in the phase 1 clinical trial of abemaciclib in previously-treated, molecularly unselected patients with advanced NSCLC [47]. Based on these results, the phase 3 JUNIPER clinical trial was designed, in which previously-treated patients with advanced NSCLC with a KRAS mutation in codon 12 or 13 were randomized to receive either abemaciclib or erlotinib as second-line treatment in patients with NSCLC with a KRAS mutation [49]. Preliminary results revealed that abemaciclib did not meet its primary endpoint of improving OS over erlotinib (median OS 7.4 months with abemaciclib, 7.8 months with erlotinib, HR 0.97 (95% CI 0.77-1.22), *p* < 0.001), although the overall response rate (8.9% in the abemaciclib arm, 2.7% in the erlotinib arm, *p* = 0.01), DCR (54.4% in the abemaciclib arm vs 31.7% in the erlotinib arm) and median PFS (3.6 months with abemaciclib, 1.9 months with erlotinib, HR 0.58 (95% CI 0.47-0.72), *p* < 0.001) were improved compared to erlotinib treatment. In our clinical study, we were not able to appreciate a correlation between KRAS status and stable disease (Table 2), likely because our sample size was small and none of the patients with adenocarcinoma had a KRAS mutation. However, we did note that cell lines with RAS mutations (KRAS, HRAS or NRAS) were more sensitive to inhibition by palbociclib, with lower IC_50_ values (Figure 3B).

In this study, our primary endpoint was response rate. Since palbociclib is now known to induce senescence, it is not surprising that treatment was associated with stable disease, and we did not observe any complete or partial responses. Similarly, in other clinical trials with CDK4/6 inhibitors in NSCLC, there is only minimal response. Therefore, future clinical trials of palbociclib should use DCR, PFS, or overall survival as an endpoint, rather than response rate. Of note, despite use of palbociclib as a single agent, the median PFS in our clinical trial is comparable to that for other effective second-line chemotherapeutic agents [50–52] and PD-1 inhibitors [53, 54]. Furthermore, the 16% rate of grade 3/4 cytopenias with palbociclib in our study (occurring in 3 of 19 patients evaluable for toxicity) was better than many available chemotherapeutic agents for second- or third-line therapy for NSCLC.

In an effort to improve the efficacy of palbociclib, we tested other agents in combination with palbociclib, and found that mTOR inhibitors were synergistic with palbociclib. Although we did not detect an increase in senescence or apoptosis with the use of everolimus, we did observe a downregulation of cyclin D1 induction following the addition of everolimus that resembles the beneficial molecular response following the combination of MEK with BRAF inhibition [55, 56].

Based on these preclinical data demonstrating synergy of palbociclib in combination with everolimus, and the demonstrated tolerability of palbociclib in patients with advanced lung cancer, a clinical trial with combined CDK4/6 and mTOR inhibition is warranted. Such a trial might also permit smaller doses of mTOR inhibitors to be used, which could potentially improve their tolerability. There is currently a phase 1 clincial trial with palbociclib and the PI3K/mTOR inhibitor gedatolisib that is enrolling patients with multiple advanced tumors including squamous cell lung cancer (NCT03065062).

## MATERIALS AND METHODS

### Study design, objectives, endpoints and eligibility

We conducted a multi-institutional phase 2 clinical trial at the University of Florida (Gainesville, FL) and the Moffitt Cancer Center (Tampa, FL) (Clinicaltrials.gov NCT01291017). The study protocol was approved by the Institutional Review Boards at each of the two participating sites. It was conducted in accordance with the Declaration of Helsinki and Good Clinical Practice guidelines. All patients gave written informed consent prior to inclusion in the study and initiation of study drug.

The primary objective was efficacy, and the secondary objectives were survival and safety. The primary endpoint was response rate using Response Evaluation Criteria in Solid Tumors (RECIST) version 1.1 criteria, and the secondary endpoints were median overall survival and progression-free survival, and determination of toxicities per Common Terminology Criteria for Adverse Events (CTCAE) version 4.0.

Patients were recruited from those already being treated at or referred to the centers. Patients 18 years of age or older with stage IV NSCLC by the 7^th^ TNM staging system, or recurrent local or locally advanced NSCLC, histologically or cytologically confirmed, were eligible to enroll. They must have been previously-treated, with any number of prior lines of systemic therapy allowed, and have documented progressive disease by RECIST v1.1. Patients were required to have an ECOG performance status of 0-2 and adequate organ and bone marrow function. Only patients whose tumors were negative for p16 expression by a CLIA-certified immunohistochemical assay were eligible. The protocol was amended during accrual to exclude patients on high-dose statins or with a history of rhabdomyolysis.

Patients were treated with palbociclib at 125 mg daily by mouth on days 1-21 of a 28-day cycle,until disease progression or 12 cycles. Palbociclib was generously provided by Pfizer, Inc. (New York, NY).

### Cell lines

All cells were cultured in RPMI 1640 medium (Sigma, St. Louis, MO) supplemented with 10% fetal bovine serum (Gibco/Thermo Fisher Scientific, Grand Island, NY) and 1% penicillin- streptomycin (Gibco/Thermo Fisher Scientific, Grand Island, NY). Human NSCLC cell lines H125 (adenocarcinoma), H226 (squamous cell carcinoma), H358 (adenocarcinoma *in situ*), H441 (adenocarcinoma), H520 (squamous cell carcinoma), H1299 (NSCLC not otherwise specified), H1650 (adenocarcinoma), H1703 (squamous cell carcinoma), H1734 (adenocarcinoma), H1792 (adenocarcinoma), H2009 (adenocarcinoma), H2347 (adenocarcinoma) and H2882 (squamous cell carcinoma) were initially isolated by the NCI-Navy Oncology Branch. RB and p16 status were validated by immunoblot analysis, and CDKN2A status was determined by PCR [4, 41]. Cell lines were tested for Mycoplasma contamination every 6 months. Mutational profiling was compiled from the Sanger Institute Catalogue of Somatic Mutations in Cancer (COSMIC) database (http://www.sanger.ac.uk/), and by IonTorrent Next Generation Sequencing (ThermoFisher Scientific, Waltham, MA) at the University of Florida.

### Reagents and antibodies

Antibody for total RB was purchased from BD Biosciences (San Jose, CA, clone C245, catalog #554136). Antibodies purchased from Cell Signaling Technology (Beverly, MA) included: total RB (clone 4H1, Catalog #9309), phospho-RB (Ser608) (#2181), phospho-AKT (Ser473) (#4060), cyclin D1 (#9309), PARP-1 (#9542), caspase 3 (#9665), caspase 9 (#9502), and phospho-p70 S6K (Thr389, #9205). β-actin antibody was purchased from Sigma Aldrich (St. Louis, MO, catalog #A1978).

Laboratory-grade palbociclib was generously provided by Pfizer, Inc. (New York, NY) and purchased from Selleck Chemicals (Boston, MA). Everolimus and pemetrexed were purchased from LC chemical (Woburn, MA). Decitabine was purchased from Sigma (St. Louis, MO). Selumetinib, flavopiridol, sunitinib, AZD5438, ponatinib, AZD4547, erlotinib, pazopanib, imatinib, PF-04691502, dacomitinib and rapamycin were purchased from Selleck Chemicals (Boston, MA). Depsipeptide was obtained from the NCI Repository in the Developmental Therapeutics Program (NSC #309132, Bethesda, MD). Stock solutions for all the agents were prepared in 100% DMSO (Sigma, St. Louis, MO) at 10mM concentrations and stored at -20°C. Stock solutions were diluted in fresh RPMI 1640 media prior to each experiment.

### Cell viability assays

For the assay, 0.85-2 × 10^3^ cells per well were counted using the N4 Cellometer (Nexelom Bioscience, Lawrence, MA) and seeded in a 96-well plate. After allowing the cells to attach overnight, they were treated with the indicated doses of palbociclib or therapeutic agent for 7 days, changing the media every 72 hours. Cell proliferation was measured using Cell Titer-Blue® Cell Viability Assay (Promega Corporation, Madison, WI). Plates were read at 560-590 nm using a SpectraMax M3 microplate reader (Molecular Devices, Sunnyvale, CA). Additional details are provided in the Supplementary Methods.

### Combination studies and determination of synergy

For the combination experiments, the IC_50_ for each drug in H358 cells was determined by the Cell Titer-Blue® Cell Viability Assay. Cells were treated for 72 hours with palbociclib (2.5 µM) and/or decitabine (9.7 nM), AZD5438 (9.7 nM), flavopiridol (0.780 µM), selumetinib (9.7 nM), or sunitinib (97nM). Cells were also treated for 7 days with palbociclib (97 nM) and/or erlotinib (9.7 nM), pemetrexed (97 nM), dacomitinib (12 nM), ponatinib (97 nM), AZD 4547 (1.56 µM), rapamycin (9.7 nM), MK2206 (19.5 nM), PF-04691502 (0.195 µM), or everolimus (9.7 nM). All concentrations used were at or near the IC_50_ doses.

Everolimus was also combined with palbociclib at various concentrations in a fixed 1:10 ratio, in the H520, H358, H2347 and H125 cell lines.

### Immunoblot analyses

Cells grown in 100 mm^3^ cell culture plates were lysed with RIPA Lysis Buffer System (Santa Cruz Biotechnology, Dallas, TX) containing Phosphatase Inhibitor Cocktail 3 (Sigma-Aldrich, St. Louis, MO). All lysates were incubated at 4°C for 20 min and centrifuged for 20 minutes at 1400 x g prior to protein quantification using BCA protein assay (Thermo Fisher Scientific, Rockford, IL). Protein (10-30 µg) was fractionated *via* 8% & 4-20% SDS-PAGE and then transferred to a nitrocellulose membrane (Thermo Fisher Scientific, Rockford, IL). All antibodies were diluted 1:1000 in 5% BSA except for β-actin antibody which was diluted 1:5000 in 5% BSA and PARP-1 antibody which was diluted 1:1000 in 5% milk. The positive control for PARP-1 was a lysate of BON cells treated for 72 hours with 10uM of PF-04554878 [57]. Rabbit or goat anti-mouse secondary antibody (Bio-Rad Laboratories, Hercules, CA) were used at 1:5000 dilution. For the detection of the protein on the immunoblot, SuperSignal Chemiluminescent Dura substrate (ThermoFisher Scientific, Rockford, IL) was used.

### Senescence assays

SA-β-Galactosidase (β -gal) staining was performed by using a Senescence β-gal staining kit (Cell Signaling Technology, Beverly, MA) according to the manufacturer's protocol. 2-5 x10^3^ cells were incubated at 37°C for 12 hours until β-gal color was detected under a bright light microscope at 100X magnification.

### Statistics

The clinical trial was powered for the primary endpoint, response rate. We assumed that a response rate lower than 10% to be a failure and a response rate over 25% to be promising. We set the significance level at 0.05 and power at 80%. Using Simon's two-stage optimum method, the total sample size was calculated as 43, with 18 patients recruited in the first stage. Assuming a dropout rate of 10%, the total sample size was calculated to be 48 and the sample size of the first stage was 20. If zero or one patient responded to the treatment at the end of the first stage, the study was to stop early. If the number of responders was two or more, the study would continue recruiting to the second stage.

For the clinical trial, the primary endpoint (i.e. response rate) was calculated as the percentage of patients with stable disease ( > 2 months). Medians of progression free survival (PFS) and overall survival (OS) with their 95% confidence intervals (CI) were estimated using the Kaplan-Meier estimator. Medians of PFS and OS were compared between patients with and without stable disease using log-rank tests.

Laboratory data were graphed as mean and standard error of the mean (SEM). Results were considered significant for *p* < 0.05. SigmaPlot software (Systat Software Inc, San Jose, CA) was used to perform four-parameter logistic regression analysis of the effect of palbociclib on cell viability in all cell lines, and to determine IC_50_ values. Data analysis for interactions between combinations of drugs was performed using the Calcusyn computational software (Biosoft, Oxford, UK), which performs multiple drug dose-effect calculations using the Median Effect Methods described by Chou-Talalay [35]. CI values below 0.8 indicate synergism; CI values between 0.8 and 1.2 represent an additive effect, and CI values greater than 1.2 represent antagonism.

## SUPPLEMENTARY MATERIALS FIGURES AND TABLES



## References

[1] Siegel RL, Miller KD, Jemal A (2018). Cancer statistics, 2018. CA Cancer J Clin.

[2] Bray F, Ferlay J, Soerjomataram I, Siegel RL, Torre LA, Jemal A (2018). Global cancer statistics 2018: GLOBOCAN estimates of incidence and mortality worldwide for 36 cancers in 185 countries. CA Cancer J Clin.

[3] America Cancer Society Non-small cell lung cancer survival rates by stage web site. http://www.cancer.org/cancer/lungcancer-non-smallcell/detailedguide/non-small-cell-lung-cancer-survival-rates.

[4] Otterson GA, Khleif SN, Chen W, Coxon AB, Kaye FJ (1995). CDKN2 gene silencing in lung cancer by DNA hypermethylation and kinetics of p16INK4 protein induction by 5-aza 2’deoxycytidine. Oncogene.

[5] Weinberg RA (1995). The retinoblastoma protein and cell cycle control. Cell.

[6] George J, Lim JS, Jang SJ, Cun Y, Ozretić L, Kong G, Leenders F, Lu X, Fernández-Cuesta L, Bosco G, Müller C, Dahmen I, Jahchan NS (2015). Comprehensive genomic profiles of small cell lung cancer. Nature.

[7] Kaye FJ (2002). RB and cyclin dependent kinase pathways: defining a distinction between RB and p16 loss in lung cancer. Oncogene.

[8] Sherr CJ, Beach D, Shapiro GI (2016). Targeting CDK4 and CDK6: From Discovery to Therapy. Cancer Discov.

[9] Cancer Genome Atlas Research Network (2012). Comprehensive genomic characterization of squamous cell lung cancers. Nature.

[10] Chen M, Voeller D, Marquez VE, Kaye FJ, Steeg PS, Giaccone G, Zajac-Kaye M (2010). Enhanced growth inhibition by combined DNA methylation/HDAC inhibitors in lung tumor cells with silenced CDKN2A. Int J Oncol.

[11] VanArsdale T, Boshoff C, Arndt KT, Abraham RT (2015). Molecular Pathways: Targeting the Cyclin D-CDK4/6 Axis for Cancer Treatment. Clin Cancer Res.

[12] Nevins JR (2001). The Rb/E2F pathway and cancer. Hum Mol Genet.

[13] Hole R (1975). Letter: undiagnosed haematuria. BMJ.

[14] Wikman H, Kettunen E (2006). Regulation of the G1/S phase of the cell cycle and alterations in the RB pathway in human lung cancer. Expert Rev Anticancer Ther.

[15] Bonelli P, Tuccillo FM, Borrelli A, Schiattarella A, Buonaguro FM (2014). CDK/CCN and CDKI alterations for cancer prognosis and therapeutic predictivity. Biomed Res Int.

[16] Fry DW, Harvey PJ, Keller PR, Elliott WL, Meade M, Trachet E, Albassam M, Zheng X, Leopold WR, Pryer NK, Toogood PL (2004). Specific inhibition of cyclin-dependent kinase 4/6 by PD 0332991 and associated antitumor activity in human tumor xenografts. Mol Cancer Ther.

[17] Klein ME, Kovatcheva M, Davis LE, Tap WD, Koff A (2018). CDK4/6 Inhibitors: The Mechanism of Action May Not Be as Simple as Once Thought. Cancer Cell.

[18] Finn RS, Martin M, Rugo HS, Jones S, Im SA, Gelmon K, Harbeck N, Lipatov ON, Walshe JM, Moulder S, Gauthier E, Lu DR, Randolph S (2016). Palbociclib and Letrozole in Advanced Breast Cancer. N Engl J Med.

[19] Asghar U, Witkiewicz AK, Turner NC, Knudsen ES (2015). The history and future of targeting cyclin-dependent kinases in cancer therapy. Nat Rev Drug Discov.

[20] Lim JS, Turner NC, Yap TA (2016). CDK4/6 Inhibitors: Promising Opportunities beyond Breast Cancer. Cancer Discov.

[21] Patnaik A, Rosen LS, Tolaney SM, Tolcher AW, Goldman JW, Gandhi L, Papadopoulos KP, Beeram M, Rasco DW, Hilton JF, Nasir A, Beckmann RP, Schade AE (2016). Efficacy and Safety of Abemaciclib, an Inhibitor of CDK4 and CDK6, for Patients with Breast Cancer, Non-Small Cell Lung Cancer, and Other Solid Tumors. Cancer Discov.

[22] Lam VK, Papadimitrakopoulou V (2018). Master protocols in lung cancer: experience from Lung Master Protocol. Curr Opin Oncol.

[23] Eisenhauer EA, Therasse P, Bogaerts J, Schwartz LH, Sargent D, Ford R, Dancey J, Arbuck S, Gwyther S, Mooney M, Rubinstein L, Shankar L, Dodd L (2009). New response evaluation criteria in solid tumours: revised RECIST guideline (version 1.1). Eur J Cancer.

[24] Oken MM, Creech RH, Tormey DC, Horton J, Davis TE, McFadden ET, Carbone PP (1982). Toxicity and response criteria of the Eastern Cooperative Oncology Group. Am J Clin Oncol.

[25] Puyol M, Martín A, Dubus P, Mulero F, Pizcueta P, Khan G, Guerra C, Santamaría D, Barbacid M (2010). A synthetic lethal interaction between K-Ras oncogenes and Cdk4 unveils a therapeutic strategy for non-small cell lung carcinoma. Cancer Cell.

[26] Klein ME, Dickson MA, Antonescu C, Qin LX, Dooley SJ, Barlas A, Manova K, Schwartz GK, Crago AM, Singer S, Koff A, Tap WD (2018). PDLIM7 and CDH18 regulate the turnover of MDM2 during CDK4/6 inhibitor therapy-induced senescence. Oncogene.

[27] Vijayaraghavan S, Karakas C, Doostan I, Chen X, Bui T, Yi M, Raghavendra AS, Zhao Y, Bashour SI, Ibrahim NK, Karuturi M, Wang J, Winkler JD (2017). CDK4/6 and autophagy inhibitors synergistically induce senescence in Rb positive cytoplasmic cyclin E negative cancers. Nat Commun.

[28] Merlo A, Herman JG, Mao L, Lee DJ, Gabrielson E, Burger PC, Baylin SB, Sidransky D (#x2032). CpG island methylation is associated with transcriptional silencing of the tumour suppressor p16/CDKN2/MTS1 in human cancers. Nat Med.

[29] Franco J, Witkiewicz AK, Knudsen ES (2014). CDK4/6 inhibitors have potent activity in combination with pathway selective therapeutic agents in models of pancreatic cancer. Oncotarget.

[30] Kwong LN, Costello JC, Liu H, Jiang S, Helms TL, Langsdorf AE, Jakubosky D, Genovese G, Muller FL, Jeong JH, Bender RP, Chu GC, Flaherty KT (2012). Oncogenic NRAS signaling differentially regulates survival and proliferation in melanoma. Nat Med.

[31] Li J, Xu M, Yang Z, Li A, Dong J (2010). Simultaneous inhibition of MEK and CDK4 leads to potent apoptosis in human melanoma cells. Cancer Invest.

[32] Wang L, Wang J, Blaser BW, Duchemin AM, Kusewitt DF, Liu T, Caligiuri MA, Briesewitz R (2007). Pharmacologic inhibition of CDK4/6: mechanistic evidence for selective activity or acquired resistance in acute myeloid leukemia. Blood.

[33] Dean JL, Thangavel C, McClendon AK, Reed CA, Knudsen ES (2010). Therapeutic CDK4/6 inhibition in breast cancer: key mechanisms of response and failure. Oncogene.

[34] Knudsen ES, Witkiewicz AK (2017). The Strange Case of CDK4/6 Inhibitors: Mechanisms, Resistance, and Combination Strategies. Trends Cancer.

[35] Chou TC (2010). Drug combination studies and their synergy quantification using the Chou-Talalay method. Cancer Res.

[36] Michaud K, Solomon DA, Oermann E, Kim JS, Zhong WZ, Prados MD, Ozawa T, James CD, Waldman T (2010). Pharmacologic inhibition of cyclin-dependent kinases 4 and 6 arrests the growth of glioblastoma multiforme intracranial xenografts. Cancer Res.

[37] Anders L, Ke N, Hydbring P, Choi YJ, Widlund HR, Chick JM, Zhai H, Vidal M, Gygi SP, Braun P, Sicinski P (2011). A systematic screen for CDK4/6 substrates links FOXM1 phosphorylation to senescence suppression in cancer cells. Cancer Cell.

[38] Kovatcheva M, Liu DD, Dickson MA, Klein ME, O’Connor R, Wilder FO, Socci ND, Tap WD, Schwartz GK, Singer S, Crago AM, Koff A (2015). MDM2 turnover and expression of ATRX determine the choice between quiescence and senescence in response to CDK4 inhibition. Oncotarget.

[39] Hidalgo M, Rowinsky EK (2000). The rapamycin-sensitive signal transduction pathway as a target for cancer therapy. Oncogene.

[40] Nelsen CJ, Rickheim DG, Tucker MM, Hansen LK, Albrecht JH (2003). Evidence that cyclin D1 mediates both growth and proliferation downstream of TOR in hepatocytes. J Biol Chem.

[41] Otterson GA, Kratzke RA, Coxon A, Kim YW, Kaye FJ (1994). Absence of p16INK4 protein is restricted to the subset of lung cancer lines that retains wildtype RB. Oncogene.

[42] Shapiro GI, Edwards CD, Kobzik L, Godleski J, Richards W, Sugarbaker DJ, Rollins BJ (1995). Reciprocal Rb inactivation and p16INK4 expression in primary lung cancers and cell lines. Cancer Res.

[43] Kratzke RA, Greatens TM, Rubins JB, Maddaus MA, Niewoehner DE, Niehans GA, Geradts J (1996). Rb and p16INK4a expression in resected non-small cell lung tumors. Cancer Res.

[44] Kaye FJ, Kratzke RA, Gerster JL, Horowitz JM (1990). A single amino acid substitution results in a retinoblastoma protein defective in phosphorylation and oncoprotein binding. Proc Natl Acad Sci U S A.

[45] Kelley MJ, Otterson GA, Kaye FJ, Popescu NC, Johnson BE, Dipaolo JA (1995). CDKN2 in HPV-positive and HPV-negative cervical-carcinoma cell lines. Int J Cancer.

[46] Okamoto A, Demetrick DJ, Spillare EA, Hagiwara K, Hussain SP, Bennett WP, Forrester K, Gerwin B, Serrano M, Beach DH (1994). Mutations and altered expression of p16INK4 in human cancer. Proc Natl Acad Sci U S A.

[47] Goldman JW, Gandhi L, Patnaik A, Rosen LS, Hilton JF, Papadopoulos KP, Tolaney SM, Beeram M, Rasco DW, Myrand SP, Beckmann RP, Kulanthaivel P, Frenzel M (2014). Clinical activity of LY2835219, a novel cell cycle inhibitor selective for CDK4 and CDK6, in patients with non-small cell lung cancer. J Clin Oncol.

[48] Mao CQ, Xiong MH, Liu Y, Shen S, Du XJ, Yang XZ, Dou S, Zhang PZ, Wang J (2014). Synthetic lethal therapy for KRAS mutant non-small-cell lung carcinoma with nanoparticle-mediated CDK4 siRNA delivery. Mol Ther.

[49] Goldman J, Mazieres J, Barlesi F, Koczywas M, Paz-Ares L (2018). A randomized phase III study of abemaciclib versus erlotinib in previously treated patients with stage IV NSCLC with KRAS mutation: JUNIPER. J Clin Oncol.

[50] Shepherd FA, Dancey J, Ramlau R, Mattson K, Gralla R, O’Rourke M, Levitan N, Gressot L, Vincent M, Burkes R, Coughlin S, Kim Y, Berille J (2000). Prospective randomized trial of docetaxel versus best supportive care in patients with non-small-cell lung cancer previously treated with platinum-based chemotherapy. J Clin Oncol.

[51] Hanna N, Shepherd FA, Fossella FV, Pereira JR, De Marinis F, von Pawel J, Gatzemeier U, Tsao TC, Pless M, Muller T, Lim HL, Desch C, Szondy K (2004). Randomized phase III trial of pemetrexed versus docetaxel in patients with non-small-cell lung cancer previously treated with chemotherapy. J Clin Oncol.

[52] Kawaguchi T, Ando M, Asami K, Okano Y, Fukuda M, Nakagawa H, Ibata H, Kozuki T, Endo T, Tamura A, Kamimura M, Sakamoto K, Yoshimi M (2014). Randomized phase III trial of erlotinib versus docetaxel as second- or third-line therapy in patients with advanced non-small-cell lung cancer: Docetaxel and Erlotinib Lung Cancer Trial (DELTA). J Clin Oncol.

[53] Brahmer J, Reckamp KL, Baas P, Crinò L, Eberhardt WE, Poddubskaya E, Antonia S, Pluzanski A, Vokes EE, Holgado E, Waterhouse D, Ready N, Gainor J (2015). Nivolumab versus Docetaxel in Advanced Squamous-Cell Non-Small-Cell Lung Cancer. N Engl J Med.

[54] Borghaei H, Paz-Ares L, Horn L, Spigel DR, Steins M, Ready NE, Chow LQ, Vokes EE, Felip E, Holgado E, Barlesi F, Kohlhäufl M, Arrieta O (2015). Nivolumab versus Docetaxel in Advanced Nonsquamous Non-Small-Cell Lung Cancer. N Engl J Med.

[55] Chapman PB, Solit DB, Rosen N (2014). Combination of RAF and MEK inhibition for the treatment of BRAF-mutated melanoma: feedback is not encouraged. Cancer Cell.

[56] Solit DB, Garraway LA, Pratilas CA, Sawai A, Getz G, Basso A, Ye Q, Lobo JM, She Y, Osman I, Golub TR, Sebolt-Leopold J, Sellers WR, Rosen N (2006). BRAF mutation predicts sensitivity to MEK inhibition. Nature.

[57] François RA, Maeng K, Nawab A, Kaye FJ, Hochwald SN, Zajac-Kaye M (2015). Targeting Focal Adhesion Kinase and Resistance to mTOR Inhibition in Pancreatic Neuroendocrine Tumors. J Natl Cancer Inst.

