# Supplementary material for: CDK4/6 inhibition stabilizes disease in patients with p16-null non-small cell lung cancer and is synergistic with mTOR inhibition
Source: Oncotarget. 2018 Dec 21;9(100):37352–66. doi: 10.18632/oncotarget.26424 (PMC6324768; doi:10.18632/oncotarget.26424)
Supplement: Supplementary file 1 [file oncotarget-09-37352-s001.pdf]

CDK4/6 inhibition stabilizes disease in patients with p16-null non-small cell lung cancer and is synergistic with mTOR inhibition

SUPPLEMENTARY MATERIALS

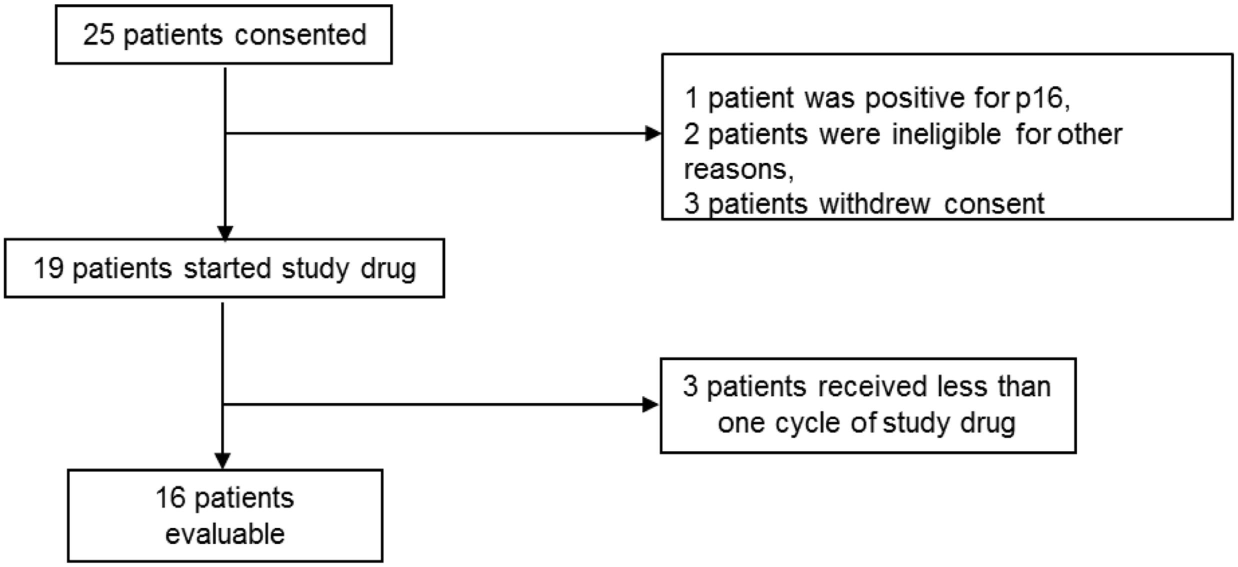

Supplementary Figure S1: Patient enrollment in the clinical trial.

Supplementary Table S1: Patient characteristics

**Age (in years)**	
Median (range)	67 (48-77)
**Gender, n(%)**	
Male	10 (53)
Female	9 (47)
**Race/Ethnicity, n(%)**	
Caucasian	17 (89)
Black/African-American	1 (5)
Latino	1 (5)
**Histology, n(%)**	
Adenocarcinoma	11 (58)
Squamous cell carcinoma	8 (42)
**Performance Status, n(%)**	
0	6 (32)
1	12 (63)
2	1 (5)
**Number of prior lines of treatment**	
Median (range)	2.16 (1-4)

Supplementary Table S2: Patient characteristics and response to treatment in patients with progressive disease.

Age	Gender	Ethnicity/ Race	Performance Status	Histology	# prior lines of therapy	EGFR status	KRAS status	ALK status	Best response	OS (months)
75	M	Caucasian	1	Adeno	3	WT	WT	WT	PD	7.6
59	F	Caucasian	1	Adeno	1	WT	WT	WT	PD	2.5
69	F	Caucasian	1	Adeno	2	WT	WT	ND*	PD	4
53	F	Caucasian	0	Adeno	1	WT	WT	WT	PD	3.4
48	F	Caucasian	0	Squamous	2	WT	WT	ND	PD	6.8
71	M	Caucasian	1	Squamous	4	ND	ND	ND	PD	7.8
55	M	Caucasian	1	Squamous	1	ND	ND	ND	PD	4.3
67	M	Caucasian	1	Squamous	2	ND	ND	ND	PD	3

M, male; F, female; WT, wildtype; ND, not determined; PD, progressive disease; OS, overall survival

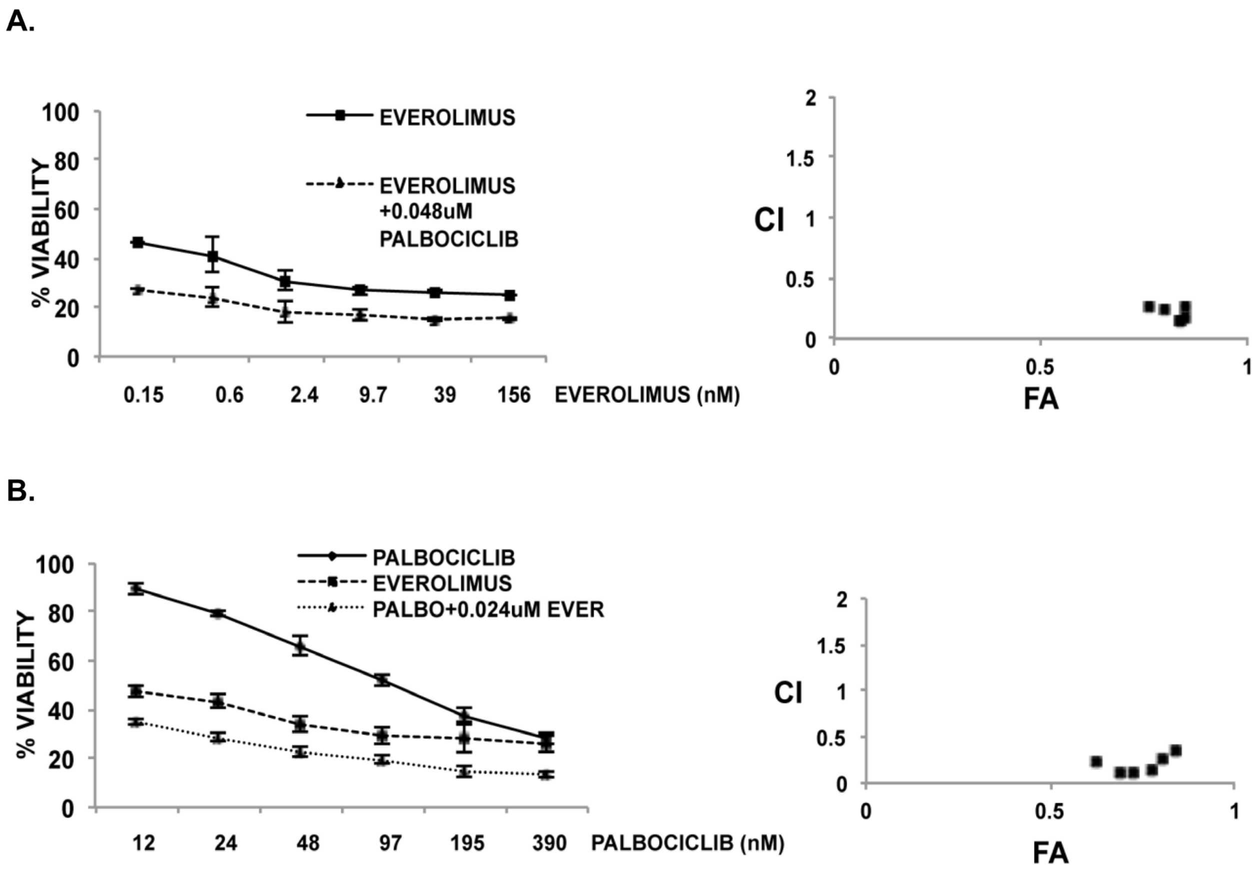

Supplementary Figure S2: Effect of the combination of everolimus and palbociclib on cell viability in H358 NSCLC cells. A. Effect of increasing concentrations of everolimus alone and in combination with a fixed concentration of palbociclib (4.8 nM). Cells were treated for seven days. CalcuSyn graph of combination index values (CI) vs Fraction Affected (FA) are shown to the right of the viability graph. **B.** Effect of palbociclib alone and in combination with a fixed concentration of everolimus (2.4 nM). Cells were treated for seven days. CalcuSyn graph of combination index values (CI) vs Fraction Affected (FA) are shown to the right of the viability graph. Data shown represent the mean ± SEM of at least 3 experiments, each performed in triplicate. Error bars indicate SEM values.

A.

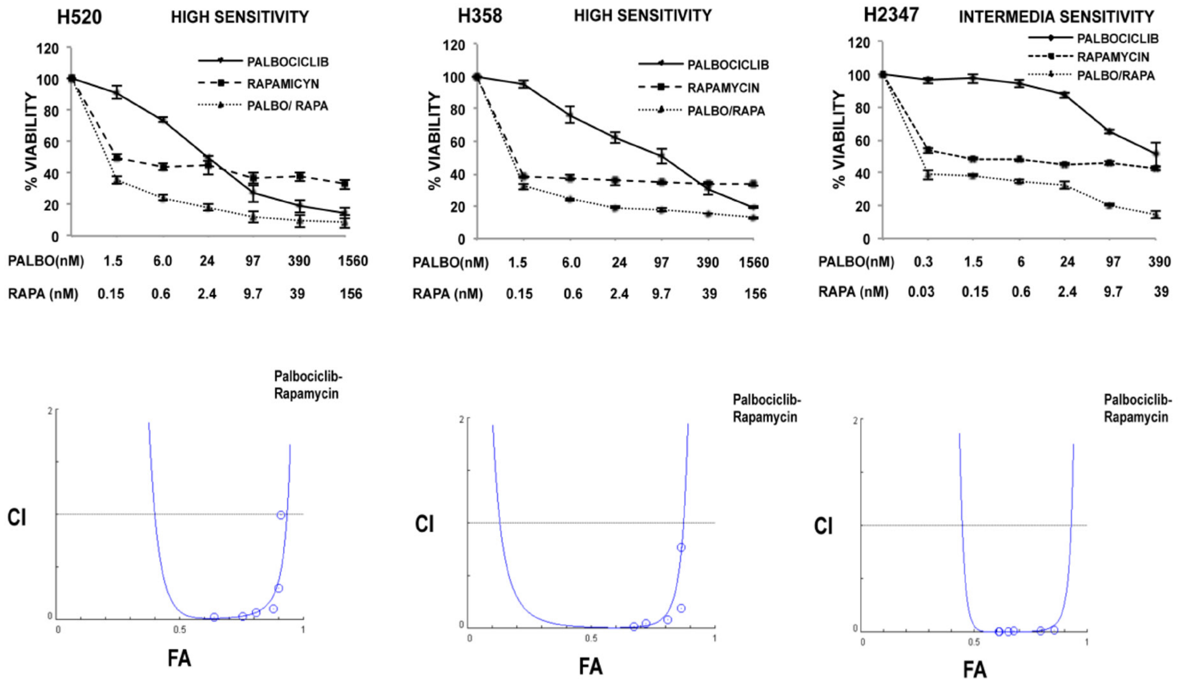

B.

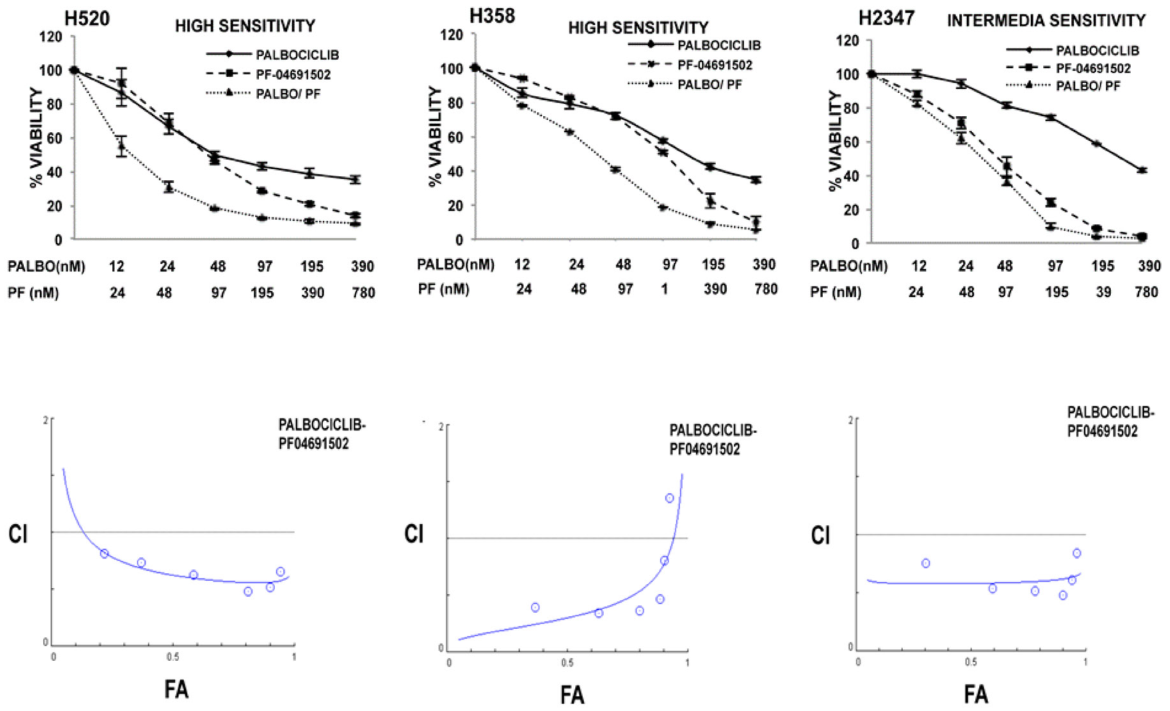

Supplementary Figure S3: Effect of palbociclib and mTOR inhibition on NSCLC cell viability. A. Effect of palbociclib and rapamycin alone and in combination at a fixed molar ratio in H520, H358 and H2347 cells. CalcuSyn graph of combination index values (CI) are shown below each viability graph. **B.** Effect of palbociclib and PF04691502 (PI3K/mTOR inhibitor) alone and in combination at a fixed molar ratio in H520, H358 and H2347 cells. CalcuSyn graph of combination index values (CI) are shown below each cell viability graph. Data shown represent the mean ± SEM of at least 3 experiments, each performed in triplicate. Error bars indicate SEM values.
